# Correction: Clinical and prognostic value of dynapenia in patients with head and neck cancer: a multicenter cohort study. VALOR group

**DOI:** 10.3389/fnut.2026.2013721

**Published:** 2026-10-12

**Authors:** María García Olivares, María Teresa Zarco-Martín, Rocío Fernández Jiménez, Montserrat Gonzalo Marín, Jose M. Romero-Márquez, Francisco Javier Vilchez López, Inmaculada Prior Sánchez, Alba Carmona Llanos, María del Carmen Roque Cuéllar, María Josefa Molina Puertas, Felisa Pilar Zarco Rodríguez, Pablo Rodríguez de Vera Gómez, Luis Miguel Luego Pérez, Gabriel Olveira, Jose Manuel García Almeida

**Affiliations:** 1 Endocrinology and Nutrition Department, Virgen de la Victoria University Hospital, Málaga, Spain; 2 IBIMA Plataforma BIONAD, Instituto de Investigación Biomédica de Málaga, Málaga, Spain; 3 Endocrinology and Nutrition Department, Quirónsalud Malaga Hospital, Málaga, Spain; 4 Department of Medicine and Dermatology, Málaga University, Málaga, Spain; 5 Endocrinology and Nutrition Department, San Cecilio University Hospital, Granada, Spain; 6 Foundation for Health Research in Eastern Andalusia - Alejandro Otero (FIBAO), Granada, Spain; 7 Granada Biosanitary Research Institute (Ibs. Granada), Granada, Spain; 8 Endocrinology and Nutrition Department, Hospital Regional Universitario de Málaga, Málaga, Spain; 9 Department of Endocrinology and Nutrition, Virgen de las Nieves University Hospital, Granada, Spain; 10 Foundation for Biosanitary Research of Eastern Andalusia-Alejandro Otero (FIBAO), Granada, Spain; 11 Department of Nutrition and Sports Sciences, Faculty of Health Sciences, International University of La Rioja, La Rioja, Spain; 12 Endocrinology and Nutrition Department, Hospital Universitario Puerta del Mar, Cádiz, Spain; 13 Biomedical Research and Innovation Institute of Cádiz (INiBICA), Cádiz, Spain; 14 Endocrinology and Nutrition Department, Jaen University Hospital, Jaen, Spain; 15 Endocrinology and Nutrition Department, Jerez de la Frontera, Hospital, Cádiz, Spain; 16 Endocrinology and Nutrition Department, Virgen del Rocio University Hospital, Sevilla, Spain; 17 Fundación para la Gestión de la Investigación en Salud de Sevilla (FISEVI), Sevilla, Spain; 18 Endocrinology and Nutrition Department, Maimonides Institute for Biomedical Research in Córdoba (IMIBIC), Reina Sofía University Hospital, Cordoba, Spain; 19 Endocrinology and Nutrition Department, Valme University Hospital, Sevilla, Spain; 20 Endocrinology and Nutrition Department, University Hospital Virgen Macarena, Seville, Spain; 21 Endocrinology and Nutrition Department, Badajoz University Hospital, Badajoz, Spain; 22 Department of Biomedical Sciences, Extremadura University, Badajoz, Spain; 23 CIBER Fisiopatalogía de la Obesidad y Nutrición (CIBERObn), Instituto de Salud Carlos III, Madrid, Spain

**Keywords:** dynapenia, handgrip strength, head and neck cancer, morphofunctional assessment, muscle strength

The article type of this article was erroneously given as: Brief Research Report. The correct article type is Original Research.

The original version of this article has been updated.

